# Development of a prognostic model to predict 90-day mortality in hospitalised cancer patients (PROMISE tool): a prospective observational study

**DOI:** 10.1016/j.lanepe.2024.101063

**Published:** 2024-10-09

**Authors:** Oriol Mirallas, Berta Martin-Cullell, Víctor Navarro, Kreina Sharela Vega, Jordi Recuero-Borau, Diego Gómez-Puerto, Daniel López-Valbuena, Clara Salva de Torres, Laura Andurell, Anna Pedrola, Roger Berché, Fiorella Palmas, José María Ucha, Guillermo Villacampa, Alejandra Rezqallah, Judit Sanz-Beltran, Rafael Bach, Sergio Bueno, Cristina Viaplana, Gaspar Molina, Alberto Hernando-Calvo, Juan Aguilar-Company, María Roca, Eva Muñoz-Couselo, Alex Martínez-Martí, Ada Alonso, Simeon Eremiev, Teresa Macarulla, Ana Oaknin, Cristina Saura, Elena Élez, Enriqueta Felip, Ángeles Peñuelas, Rosa Burgos, Patricia Gómez Pardo, Elena Garralda, Josep Tabernero, Sònia Serradell, Sònia Servitja, David Paez, Rodrigo Dienstmann, Joan Carles

**Affiliations:** a Medical Oncology Department, Vall d’Hebron Hospital Campus and Vall d’Hebron Institute of Oncology (VHIO), 08035, Barcelona, Spain; b Medical Oncology Department, Hospital de la Santa Creu I Sant Pau, 08041, Barcelona, Spain; c Medical Oncology Department, Hospital del Mar, 08003, Barcelona, Spain; d Oncology Data Science (ODysSey) Group, Vall d'Hebron Institute of Oncology (VHIO), 08035, Barcelona, Spain; e Nutritional Support Unit, Vall d’Hebron Hospital Campus, 08035, Barcelona, Spain; f Nursing Department, Vall d’Hebron Hospital Campus, 08035, Barcelona, Spain

**Keywords:** Prognostic factors, Hospital oncology service, 90-day mortality, PROMISE tool, LASSO method

## Abstract

**Background:**

Prognostic factors for ambulatory oncology patients have been described, including Eastern Cooperative Oncology Group (ECOG), tumor stage and malnutrition. However, there is no firm evidence on which variables best predict mortality in hospitalized patients receiving active systemic treatment. Our main goal was to develop a predictive model for 90-day mortality upon admission.

**Methods:**

Between 2020 and 2022, we prospectively collected data from three sites for cancer patients with hospitalizations. Those with metastatic disease receiving systemic therapy in the 6 months before unplanned admission were eligible to this study. The least absolute shrinkage and selection operator (LASSO) method was used to select the most relevant factors to predict 90-day mortality at admission. A multivariable logistic regression was fitted to create the PROgnostic Score for Hospitalized Cancer Patients (PROMISE) score. The score was developed in a single-center training cohort and externally validated.

**Findings:**

Of 1658 hospitalized patients, 1009 met eligibility criteria. Baseline demographics, patient and disease characteristics were similar across cohorts. Lung cancer was the most common tumor type in both cohorts. Factors associated with higher 90-day mortality included worse ECOG, stable/progressive disease, low levels of albumin, increased absolute neutrophil count, and high lactate dehydrogenase. The c-index after bootstrap correction was 0.79 (95% CI, 0.75–0.82) and 0.74 (95% CI, 0.68–0.80) in the training and validation cohorts, respectively. A web tool (https://promise.vhio.net/) was developed to facilitate the clinical deployment of the model.

**Interpretation:**

The PROMISE tool demonstrated high performance for identifying metastatic cancer patients who are alive 90 days after an unplanned hospitalization. This will facilitate healthcare providers with rational clinical decisions and care planning after discharge.

**Funding:**

Merck S.L.U., Spain.

**Research in context**

**Evidence before this study**

Spain is currently facing major challenges in terms of the economic sustainability of its public healthcare system, particularly in view of rising life expectancy and the increase in chronic diseases such as cancer. Unplanned hospitalization represents a heavy psychological and financial burden to the patient, family, and society. Survival estimates are an important element of decision-making in oncology care. With more accurate assessment of prognosis, the oncologist is better placed to offer adequate and adapted advanced care, which is critical for cancer patients with metastatic disease who are receiving active systemic treatment.

There are several prognostic factors for predicting the survival outcomes of ambulatory patients that are often included in the planning of a therapeutic management strategy. Only one small French study has described a clinical prognostic profile beyond ECOG for hospitalized cancer patients, but it was designed for palliative patients (less than 6 months survival). However, to our knowledge, predicting outcomes for advanced cancer patients who experience unplanned hospitalization is limited to the elderly population and patients treated with chemotherapy; it therefore remains to be studied in the broader cancer population.

After research on Pubmed (2024/02/01) using the key words “Cancer” and “Hospitalization” and “Prognostic tool”, we did not find any article that describe a prognostic score for hospitalized patients with advanced cancer on treatment.

**Added value of this study**

This study adds value to the management of oncologic patients as it offers physicians a new prognostic tool to implement based on a multicentric real-world experience of hospitalized cancer patients under active and contemporary oncological treatments. Our prospective multicentric study, comprising 1009 metastatic oncologic patients undergoing systemic treatment, identifies ECOG performance status, oncologic treatment response, LDH, neutrophil count, and albumin levels upon admission as prognostic indicators for 90-day mortality, yielding a PROMISE score with a c-index of 0.79.

**Implications of all the available evidence**

The integration of the PROMISE score into routine clinical practice, based on prospective, real-world data of hospitalized oncologic patients undergoing systemic treatment, provides physicians with an improved decision-making tool, thereby fostering optimized patient outcomes.

## Introduction

Cancer will soon rank as the leading cause of death worldwide, representing the greatest barrier to increasing life expectancy in the mid-21st century.^1^^,^^2^ Cancer patients frequently require unplanned hospitalized care as a result of treatment toxicities, infections, along with complications from the cancer itself.^3^^,^^4^

Oncologic treatment is a delicate balance of prolonging survival and maximizing end-of-life quality for hospitalized patients.^5^ However, hospitalization contributes to the high cost of cancer care and is a stressful experience for both the patient and the caregiver.^6^, ^7^, ^8^ Thus, hospitalization is often counterproductive to improving patient health and is increasingly recognized as poor-quality cancer care.^9^, ^10^, ^11^ Furthermore, inpatient hospitalizations bear a higher financial burden compared to outpatient support.^12^, ^13^, ^14^ Despite this evidence, hospitalization during the oncologic process is inevitable and must be optimized to improve outcomes.

Survival outcomes for cancer patients are influenced by a multitude of factors including tumor type and stage, oncogenic drivers, patients’ general status, and clinicopathologic factors. Validated prognostic factors for survival outcomes in the outpatient setting have been well established, with many international management guidelines, such as the European Society for Medical Oncology and the National Comprehensive Cancer Network, recommending treatments based on disease stage and Eastern Cooperative Oncology Group (ECOG) performance status.^15^^,^^16^ However, current estimators of mortality among metastatic cancer patients with unplanned hospitalizations lack solid evidence. The clinical status of patients undergoing an acute illness or complication during the oncologic treatment is different from stable ambulatory cancer patients. Identifying these predictors at admission will help inform clinical interventions and avoid unnecessary procedures in situations lacking clear treatment benefit, reducing the cost, and improving the quality of care.

The main objective of our study was to determine the predictors of mortality of an individual patient at the admission of an unplanned hospitalization. Integrating multiple clinical factors into a model that can serve as a practical tool for physicians will allow appropriate adjustment of medical interventions during hospitalization and post-discharge, based on the initial individual patient's outlook.

## Methods

### Study design and patient population

This prospective multicentric study was performed in three reference cancer centers in Spain. Data were obtained from the database called PLANTOLOGY (which means “ward” in Spanish), which includes prospective data collected at the time of admission of all cancer patients undergoing hospitalization for any reason. Data included demographics, comorbidities classified according to the Charlson Comorbidity Index (CCI), disease characteristics, cancer treatments, and laboratory results. Tumor response, number of prior treatment lines and survival outcomes were collected from medical records. Laboratory results from different hospitals were converted to uniform units to standardize the data. The standard procedure for assessing pain involved describing and categorizing its location using the Visual Analog Scale and the Common Terminology Criteria for Adverse Events v5.0 upon admission of patients to the emergency department. All admissions for pain, irrespective of severity or location, were collectively analyzed and related to the patients' diagnoses. To be eligible, patients had to be at least 18 years old, have an advanced/metastatic histologically confirmed solid tumor, have received systemic anticancer therapy for at least 6 months prior to hospitalization and have undergone an unplanned hospitalization lasting at least 24 h in a regular ward or the emergency department. All variables were selected based on known prognostic factors in the outpatient setting and exploratory analyses published in the inpatient setting to provide a comprehensive assessment of all factors that may predict the outcome of hospitalized cancer patients.^15^^,^^16^ The training cohort comprised consecutive patients hospitalized between March 2020 and February 2022 at the Vall d’Hebron University Hospital. Outcomes were validated in an external cohort of consecutive patients hospitalized between January 2021 and February 2022 in two additional cancer centers (Sant Pau Hospital and Mar Hospital). The final model included only the first admission for each patient, excluding any subsequent admissions. However, the total number of admissions was quantified, and the time intervals between consecutive admissions were characterized. The study is registered at ClinicalTrials.gov (NCT05534178). It was approved by the independent Ethics Committee at each center and was conducted in accordance with Spanish and European regulatory authorities' requirements. All patients provided written informed consent prior to study entry. Due to COVID-19 prevention measures, most COVID-19 infected patients during 2020 and 2021 did not consent; we therefore excluded all COVID-19-related admissions.

### Statistical analysis

To address the primary objective of the study, univariable and multivariable logistic regression models were constructed to develop a predictive algorithm for estimating the probability of 90-day mortality from admission in the training cohort, which was termed the Prognostic Score for Hospitalized Cancer Patients (PROMISE score). The model selected the most relevant variables using the LASSO (Least Absolute Shrinkage and Selection Operator) method^17^ with lambda 1 standard error in the training cohort. The LASSO method helps to choose the most relevant variables while simplifying the model. The linearity assumption of continuous predictors among the final selected variables was relaxed using restricted cubic splines via the rms R package. The predictions for each patient were obtained using the coefficients of the multivariable logistic model, which was constructed with variables selected by LASSO and incorporated cubic splines for continuous variables (Supplementary Figure S1A).

To validate the model internally, resampling with bootstrap and optimism corrected was performed,^17^ which involves repeated sampling from the original dataset with replacement, creating multiple datasets of the same size as the original. This process allows for estimating the variability of the model's performance and assessing the presence of overfitting in the training cohort, by calculating the area under the curve (AUC) 1000 times using variations of the data through bootstrap resampling, comparing it with the original AUC. Additionally, a calibration plot analysis was conducted to assess the agreement between observed and predicted outcomes, providing insight into the model's calibration or accuracy.^18^

Moreover, the model was evaluated in the external validation cohort using AUC with a 95% Confidence Interval (95% CI), calculated through 2000 bootstrap resamples. The evaluation metrics also included Negative Predictive Value (NPV), Positive Predictive Value (PPV), specificity, sensitivity, and accuracy. These metrics were computed with high risk representing 90-day mortality and low risk representing non-90-day mortality, while the intermediate risk, comprising patients with less distinct characteristics and therefore difficult to classify, was kept separate from the evaluation. A calibration plot of the external validation was constructed. The cutoff for group classification was determined by the tertiles method (Supplementary Figure S1B), in which the training cohort was divided into three groups with similar sample sizes. Median follow-up was calculated with the reverse Kaplan–Meier method from the time of admission. Overall survival (OS) was calculated from the time of admission and was estimated with the Kaplan–Meier method and a Cox model was used to detect differences; hazard ratio (HR) with 95% confidence intervals (CI) and P-values were reported. The CCI was determined as described.^19^ Missing at random values were filled in for the variables incorporated in the PROMISE score using the Multivariate Imputation via Chained Equations (MICE) method.^20^ MICE impute missing values based on the observed values of other variables, ensuring that the imputed values maintain the underlying relationships within the dataset. R software 4.2.2 was used to conduct statistical analysis. Statistical significance levels were two-sided, and the significance threshold was defined as P < 0.05.

### Role of the funding source

The development of this publication was supported by Merck S.L.U., Spain, through a grant for the writing of an independent medical publication. Thus, the funder of the study had no role in the study design, data collection, analysis, interpretation of the data, and writing of the report.

## Results

### Patient population

**Fig. 1:**
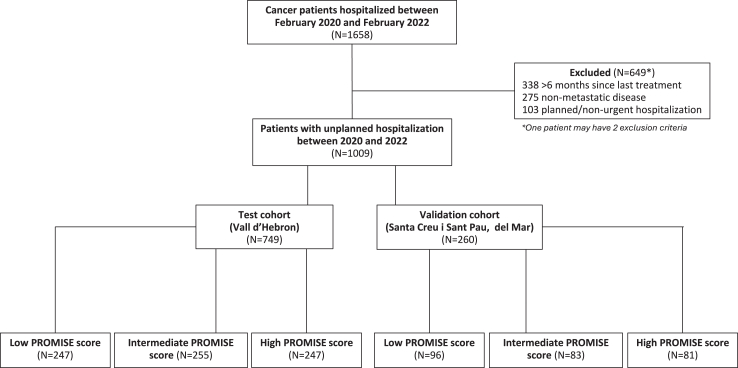
Population flow chart.

Among 1658 patients in the PLANTOLOGY database, 1009 with metastatic disease who had received treatment for their primary cancer at least once during the 6 months before unplanned hospitalization were eligible (Fig. 1). The training cohort was composed of 749 patients and the external validation cohort included 260 patients.

In the training cohort, the median patient age was 65 years (IQR 56–72), 51% of patients were female, 43% had an ECOG performance status ≥2, 43% were treated in a clinical trial, and the median CCI was 8 (IQR 7–9) (Table 1). Patients had been diagnosed with a median of 22 months before hospitalization and 65% of patients had metastatic disease at diagnosis. Imputed and missing values from the training cohort are described in Supplementary Table S1. The training and validation cohorts were generally similar in terms of demographics, baseline and disease characteristics; the main differences (≥10% difference) were a lower proportion in the training cohort of males (49% vs 62%, respectively), patients with ECOG ≥2 (43% vs 59%), smokers (48% vs 61%), patients receiving chemotherapy (56% vs 67%), whereas more patients in the training cohort were being treated in a clinical trial (42% vs 7%; Table 1). The most frequent tumor types in both the training and validation cohorts were lung (23% and 28%, respectively), colorectal (15% and 12%, respectively), and breast (12% and 9%, respectively; Supplementary Figure S2A).

**Table 1:** Patient demographic and disease characteristics, hospitalization and clinical status in the training and validation cohorts.

	Training cohort (N = 749)	Validation cohort (N = 260)
Age in years, median (IQR)	65 (57–73)	68 (58–76)
Gender		
Male	369 (49%)	162 (62%)
Female	380 (51%)	98 (38%)
ECOG PS		
0–1	428 (57%)	106 (41%)
2–4	321 (43%)	154 (59%)
Smoker	360 (48%)	159 (61%)
Alcohol intake^a^	68 (9%)	18 (7%)
Dyslipidemia	221 (30%)	95 (37%)
Hypertension	274 (37%)	124 (48%)
Chronic cardiac disease^b^	56 (7%)	34 (13%)
Albumin g/dL, median (IQR)	3.3 (2.8–3.7)	3.2 (2.8–3.6)
LDH UI/L, median (IQR)	268 (203–401)	226 (71–3703)
WBC x10^9^/L, median (IQR)	8.0 (5.2–11.7)	7.37 (4.4–11.5)
Charlson Comorbidity Index, median (IQR)	8 (7–9)	9 (7–10)
Time since diagnosis in months, median (IQR)	22.2 (8.0–51.6)	17.7 (5.2–46.4)
Prior cancer to the cancer currently being treatment	76 (10%)	40 (15%)
Disease stage at diagnosis		
I-III	249 (35%)	111 (43%)
IV	466 (65%)	149 (57%)
N lines prior anticancer therapy, median (IQR; minimun/maximum)	2 (1–3; 1/12)	2 (1–3; 1/10)
Anticancer therapy during 6 months prior to hospitalization^c^		
Chemotherapy	411 (56%)	173 (67%)
Immunotherapy	155 (21%)	38 (15%)
Targeted therapy	107 (15%)	27 (10%)
Radiotherapy	19 (3%)	5 (2%)
Other^d^	42 (6%)	22 (8%)
Anticancer therapy in a clinical trial	316 (42%)	17 (7%)
Response at last tumor assessment^e^		
Complete/partial response	102 (18%)	48 (24%)
Stable disease	119 (21%)	38 (19%)
Progressive disease	343 (61%)	116 (57%)
Duration of hospitalization in days, median (IQR)	9 (6–14)	9 (6–14)
Thrombosis during hospitalization	45 (6%)	4 (2%)
Opioid use prior to admission^e^	214 (34%)	89 (35%)
Opioid use at discharge^e^	263 (41%)	112 (45%)

ECOG PS, Eastern Cooperative Oncology Group (ECOG) performance status.

a ≥4 standard drinks/daily.

b Previously reported acute myocardial infarction, valvopathy, cardiac insufficiency (excludes arrhythmia).

c Patients could receive more than one type of therapy.

d Hormone therapy, gene therapy, surgery, cell therapy.

e Missing data; percentage calculated based on patients with data.

### Hospitalization and survival outcomes

The most frequent symptoms leading to unplanned hospitalization in both the training and validation cohorts were pain (20% and 17%, respectively), fever (14% and 27%, respectively), and dyspnea (15% and 12%, respectively; Supplementary Figure S2B). The diagnosis underlying the reason of admission was analyzed; infection justified fever in 52% of the patients in the training cohort and 46% in the validation cohort, disease progression justified approximately one-third of the hospitalizations for pain in both cohorts, and pleural effusion justified 21% of dyspnea in the training cohort and respiratory tract infection justified 36% of dyspnea in the validation cohort (Supplementary Table S2). Unplanned hospitalizations lasted a median of 9 days (IQR 6–14) for both the training and the validation cohorts.

After a median follow-up of 16 months (95% CI 13.4–17.9) for the training cohort and 12.1 months (95% CI 10.9–13.4) for the validation cohort, the 90-day mortality rate after discharge was 41.9% in the training cohort and 43.1% in the validation cohort. Median OS after discharge in the training cohort was 5.4 months (95% CI 4.6–6.7) and 4.7 months (95% CI, 3.1–6.5) in the validation cohort. A total of 228 patients (22.5%) experienced a second admission, with a median time of 48 days (IQR 23–116) after discharge, most often for the same reason as their previous admisison. Of these, 48 patients had a third admission, and only 10 patients experienced a fourth admission (Supplementary Figure S3).

### PROMISE score composition and performance

**Fig. 2:**
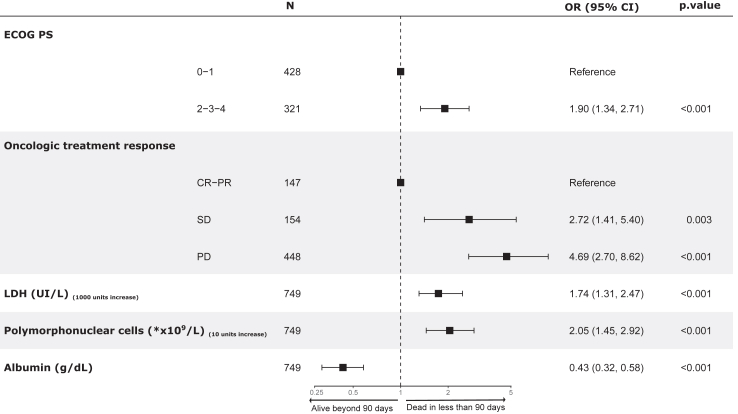
Multivariate analysis of PROMISE score variables influencing 90-day mortality after unplanned hospitalization in the training cohort. ∗Legend: The variables ECOG and response on current treatment (CR-PR, SD, PD) were used as categorical variables; the serum levels of lactate dehydrogenase (LDH) in U/L, polymorphonuclear cells count (PMN) in ∗×10ˆ9/L, and albumin in g/dL were used as continuous variables in our model.

**Fig. 3:**
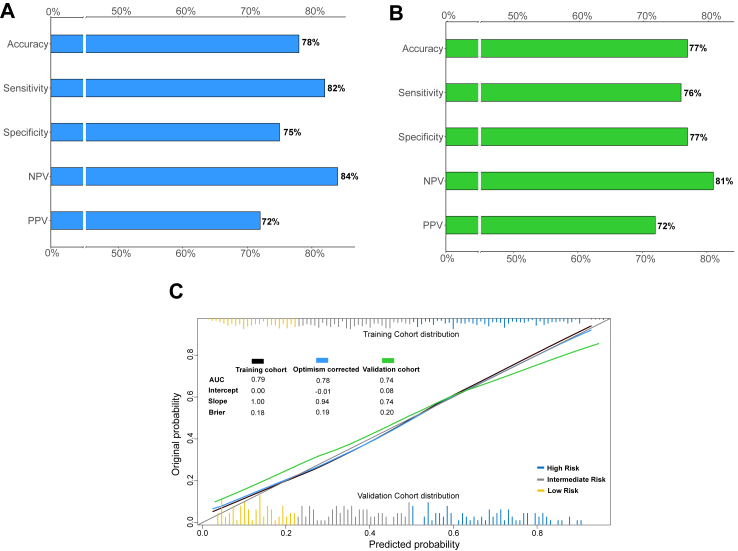
Barplots depict discriminative performance metrics of the PROMISE score in the training cohort (A) and validation cohort (B). Calibration plots display the observed vs predicted outcomes in the training cohort before and after bootstrap optimism correction, as well as in the validation cohort (C).

The PROMISE score was developed in the training cohort with variables selected from the univariate (Supplementary Figure S4) and multivariable logistic prognostic model (Fig. 2). The variables retained were ECOG performance status (0–1, 2–4) and response on current therapy (complete or partial response, stable disease, progressive disease) as categorical variables; the serum levels of lactate dehydrogenase (LDH) in U/L, polymorphonuclear cells count (PMN) in ∗×10ˆ9/L, and albumin in g/dL as continuous variables (Fig. 2). The score was well calibrated in the training and validation cohorts, indicating that the probability values provided by this score are highly reliable (Fig. 3C). The slope and intercept were 0.94/−0.01 and 0.74/0.08 in the training cohort after correcting for optimism and validation cohort, respectively. Bootstrap analysis showed low optimism (less than 1%). The AUC metrics (c-index) were 0.78 (95% CI, 0.74–0.81) after correcting for optimism in the training cohort and 0.74 (95% CI, 0.68–0.80) in the validation cohort (Fig. 3C). The PROMISE score was also applied to the most common tumor types — lung, breast, colorectal, and others — with good performance (C-index for lung 0.78 (95% CI, 0.71–0.85); breast 0.81 (95% CI, 0.71–0.90); colorectal 0.82 (95% CI, 0.75–0.90), and others 0.79 (95% CI, 0.72–0.85)) [Supplementary Table S3].

### Clinical utility of the PROMISE score

For clinical simplification and implementation purposes, the PROMISE score was categorized in risk groups using tertiles: high risk (score >53.0%), intermediate risk (score >27.3%), and low risk (score ≤27.3%). A total of 247 patients (33%) from the training cohort and 81 patients (30%) from the validation cohort were classified as high-risk (Fig. 1). The PROMISE score performance shows a negative predictive value of 84% (207/247), sensitivity of 82% (179/219) and overall accuracy of 78% (386/494) in the training cohort, and a negative predictive value of 81% (78/96), sensitivity of 76% (58/76) and overall accuracy of 77% (136/177) in the validation cohort (Fig. 3A and B).

**Fig. 4:**
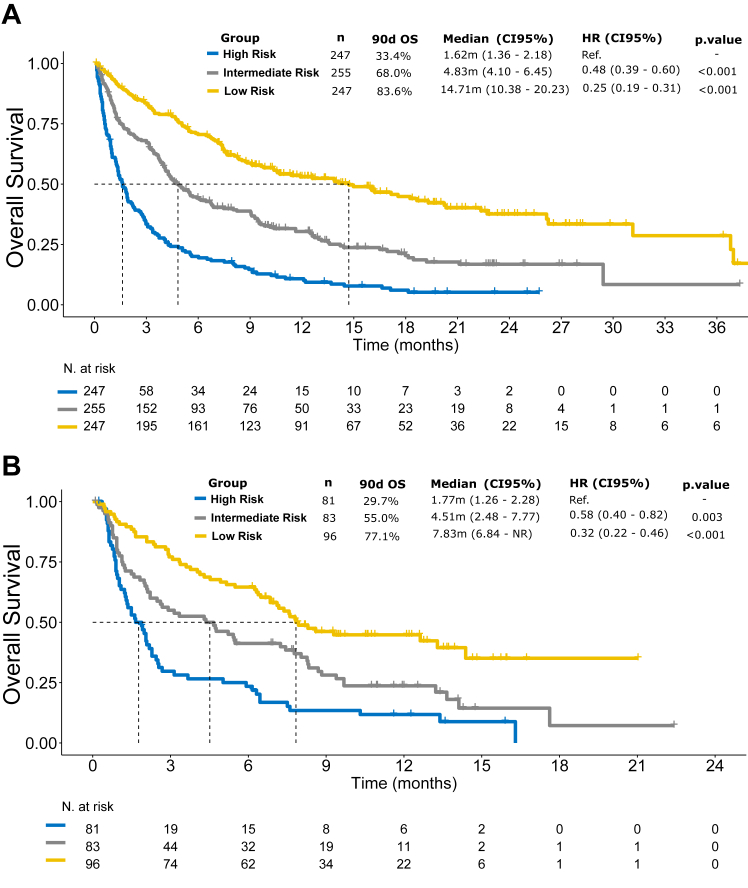
Kaplan–Meier curves for overall survival, showing for 90-day survival rates, by risk group according to high, intermediate, or low PROMISE score, in the training (A) and validation (B) cohorts.

Patients were stratified into three groups based on the PROMISE score for OS in both the training and validation cohorts, as shown in the Kaplan–Meier curves (Fig. 4). The application of the threshold of the PROMISE score in the training cohort demonstrated a survival rate at 90 days of 84% (95% CI 79–88) in patients with a low PROMISE score, 68% (95% CI 62–74) in patients with an intermediate PROMISE score, and a 33% (95% CI 27–41) in patients with a high PROMISE score (HR 0.25, 95% CI 0.19–0.31; Fig. 4A). When the PROMISE threshold was applied to the validation cohort, a survival rate at 90 days of 77% (95% CI 69–86) was seen in patients with a low PROMISE score, 55% (95% CI 45–67) in patients with intermediate PROMISE score, and a 30% (95% CI 20–43) in patients with a high PROMISE score (HR 0.32, 95% CI 0.22–0.46; Fig. 4B). The PROMISE score was also applied to lung, breast, and colorectal cancer to conduct survival analysis to assess its prognostic value for each particular risk group (Supplementary Figure S5).

In addition, we developed a web application (https://promise.vhio.net/) that enables the calculation of the PROMISE score using the information for the seven above-mentioned variables, to facilitate clinical deployment of the model and future validation in a larger cohort.

## Discussion

Unplanned hospitalization represents a heavy psychological and financial burden to the patient, family, society, and improving the outcome of this scenario has obvious benefits. Our population with primarily lung, gastrointestinal and gynecological cancers had poor prognosis overall. With more than half of the patients diagnosed with stage IV disease, an ECOG of at least two, and receiving multiple prior therapies, unplanned hospitalizations are expected. Survival estimates are an important element of decision-making in oncology care. With more accurate assessment of prognosis, the oncologist is better placed to offer adequate and adapted advanced care, which is critical for cancer patients with metastatic disease who are receiving active systemic treatment. Several prognostic factors exist for predicting survival outcomes for the ambulatory patient and are often incorporated into the planning of a therapeutic management strategy. However, to our knowledge, predicting outcomes for advanced cancer patients who experience unplanned hospitalization is limited to the elderly population; it therefore remains to be studied in the broader cancer population.^21^, ^22^, ^23^

In this prospective study, we developed a prognostic model to fill an important gap in our knowledge and clinical practice in this commonly encountered scenario. The PROMISE score helps address prognostic uncertainty for early mortality among pan-cancer patients experiencing unplanned hospitalization while undergoing systemic therapy. The score is composed of readily available or easily measurable variables during routine clinical care. In our model, a worse ECOG performance status, stable/progressive disease on the patient's current therapy, and abnormal values for routine biological analyses (LDH, PMN, and albumin) were associated with higher 90-day mortality rates after univariable analyses and remained significant in the multivariate analysis after LASSO selection. These factors combined reflect more aggressive disease with higher tumor burden, inflammation due to increased PMN, and patient frailty. The high negative predictive value of 84% (207/247), high c-index (79% (95% CI, 75–82)) with an accuracy of 78% (386/494) of the PROMISE score in the training cohort, with a comparable AUC curve in the validation cohort confirm its power as a tool for physicians to accurately discriminate patients most likely to benefit from more aggressive, intensive medical treatment.

Comparison with studies assessing predictors of survival in the context of unplanned hospitalizations in advanced cancer patients is challenging, in part due to the limited published data and variations in settings (heterogeneous disease stages, tumor types, treatments, endpoints, etc.). Available studies focus on geriatric patients, small unicentric studies with treatments mainly based on chemotherapy that do not reflect the current therapeutic landscape.^22^, ^23^, ^24^, ^25^, ^26^ Preliminary results from a study in patients with a median age of 84 years reported higher CCI, poorer ECOG performance status and disease progression to be associated with in-hospital mortality.^27^ A small French study of 177 patients from a single institution with no active treatment and in a palliative stage reported that albumin, LDH and Karnofsky performance status classified hospitalized palliative patients into three prognostic groups.^21^ However, none of the studies described a cross-cancer cohort of more than 1000 patients undergoing active treatment, multiple types of treatments including immunotherapies or targeted agents and novel oncologic treatments, clinical and laboratory parameters, and none have previously developed an easy-to-use web tool with such strong predictive power for hospitalized cancer patients.

Hospitalization is inevitable during the oncology process, and so our study gives oncologists a simple and accessible tool, such as the PROMISE score, to quickly identify patients with the best chance of survival without having to perform additional investigations (an important consideration in terms of quality of life and cost), and to initiate more aggressive therapeutic interventions for those with a favorable prognosis. Hypothetically, for patients with high risk of early mortality based on the PROMISE score, starting supportive care management during hospitalization may improve both survival and quality of life. Care can instead be focused on providing nutritional support and community-based palliative care interventions which have proven to be effective in improving survival and quality of life, as well as reducing costs due to avoidable prolonged and repeated hospitalizations for terminally ill patients.^28^, ^29^, ^30^

This study has several limitations. Firstly, the analysis cohorts represent a Spanish population with a range of solid tumor types, variable length of time since diagnosis and stages; the training population was from a single institution and the validation cohort included a smaller number of patients from another two institutions with limited access to clinical trials, which may limit the generalization of the results. Our study excluded COVID-19-related admissions due to stringent prevention measures during the pandemic hindered the ability to obtain informed consent from these patients, thereby limiting the results to oncologic patients who are not infected with COVID-19. Patients with lung cancer accounted for a notably higher proportion of the study population compared to other tumor types, and there were some differences between the training and validation populations, including ECOG performance status, clinical trial inclusion, and response to current treatment. The strengths of our study may neutralize some limitations. Data were collected prospectively, and our model was developed in a large population of more than 1000 cancer patients with characteristics that are a good match to real-life cancer treatment settings, with patients having a high CCI and advanced disease (median two years since diagnosis). In addition, patients with all types of oncological treatments were included, not just chemotherapy as previous studies reported.^4^ Our model was externally validated using data from two hospitals, and the PROMISE score retained its performance in different tumor types, namely lung, breast, and colorectal. Despite the heterogeneous population due to a pan-cancer scenario and some differences in characteristics between cohorts, the model had a very high accuracy, showing that the score PROMISE will likely be widely applicable. The variables selected are objective and routinely collected in most hospitals, unlike more subjective variables that require patient-reported outcomes and additional assessments during hospitalization. Finally, by developing a web application with a user-friendly interface, we offer physicians an easy and accessible means of implementing the model to quantify the risk of early mortality in hospitalized cancer patients and thereby make more informed decisions in routine care.

To conclude, the PROMISE score integrates readily available clinical and laboratory factors at admission to accurately predict 90-day mortality after an unplanned hospitalization in advanced cancer patients receiving active treatment. The PROMISE score will allow physicians to identify patients most likely to benefit from more intensive care or less aggressive interventions. We believe the PROMISE score offers a new and accessible tool for accurate decision-making by physicians and hospitals when a cancer patient is undergoing active treatment and requires unscheduled hospitalization.

## Contributors

OM, RD, and JC designed and wrote the first draft of the report. JAC, MR, AA, SE, TM, AO, CS, EF, AP, EMC, AMM, EE, RB, PG, EG, JT, SS, SS, DP, RD, and JC provided the study materials or patients toward the study. OM, BM, KSV, JRB, DGP, DLV, CST, LA, AP, RB, FP, JMU, AR, JSB, RB, SB, CV, and GM collected and assembled the data. AP, RB, and VN did the software development. VN, GV, and RD performed the data analysis and interpretation. OM, CST, KSV, and VN accessed and verified all the data. Lastly, all auhors critically reviewed and approved the manuscript.

## Data sharing statement

Deidentified patient data from this study can be made available to qualified investigators who provide a methodologically sound research proposal and sign a data access agreement. Please email oriolmirallas@vhio.net for information. The study protocol, statistical analysis plan, and informed consent form will also be made available upon request. Data will be shared via a secure online platform, REDcap and https://github.com/VHIO-Odyssey/PROMISE.

## Declaration of interests

OM reports writing aid from Merck and Roche, and travel aid from Almirall, Kyowa Kirin, and Recordati. JR reports payment or honoraria for lectures from Merck and LEO Pharma, and travel aid from Adamed and LEO Pharma. DG reports payment or honoraria for lectures from LEO Pharma. RB is currently employed by AstraZeneca. GV reports consulting fees from Reveal Genomics, and payment or honoraria for lectures from MSD, Pierre Fabre, Pfizer, and GSK. SB reports payment or honoraria for lectures from Pfizer, and travel aid from MSD. AH reports grants for research support from Gilead, and payment or honoraria for lectures at the TTCC and THNO congresses. MR reports payment or honoraria for lectures from ROVI. EM reports consulting fees from BMS, MSD, Novartis, Sanofi, Pierre Fabre, Regeneron, Inmunocore, and Roche, payment or honoraria for lectures from BMS, MSD, Novartis, Sanofi, Pierre Fabre, Regeneron, and Inmunocore, and travel aid from MSD and Novartis. AM reports consulting fees from AstraZeneca, BMS, Roche, and MSD, payment or honoraria from AstraZeneca, BMS, Roche, participation as a steering committee member for AstraZeneca, travel aid from AstraZeneca, BMS, and Roche, and participation on the advisory board for AstraZeneca, MSD, and BMS. TM reports grants from MSD, Novocure, QED Therapeutics, Roche, Sanofi-Aventis, Servier, Zymeworks, consulting fees from Ability Pharmaceuticals SL, Arcus Bioscience Inc., AstraZeneca, Basilea Pharma, Baxter, BioLineRX Ltd, Celgene, Eisai, Incyte, Ipsen Bioscience Inc., payment or honoraria from Janssen, Lilly, Esteve, Daïchi, Biontech, Novartis, Jazz Pharmaceuticals, and travel aid from Servier, AstraZeneca, Sanofi, Incyte, Lilly, MSD, and Roche. AO reports consulting fees from AstraZeneca, Clovis Oncology, Corcept Therapeutics, Deciphera Pharmaceuticals, Daiichi Sankyo, Debiopharm International, Eisai, Exelixis, F. Hoffmann-La Roche, Genmab, GSK, ImmunoGen, Itheos, MSD, Mersana Therapeutics, Myriad Genetics, Novocure, OncoXerna Therapeutics Inc., PharmaMar, Regeneron, Sattucklabs, Seagen/Pfizer, Sutro Biopharma, and Zentalis; travel aid from AstraZeneca, PharmaMar, and Roche, and participation on the advisory board for AstraZeneca, Clovis Oncology, Corcept Therapeutics, Deciphera Pharmaceuticals, Daiichi Sankyo, Debiopharm International, Eisai, Exelixis, F. Hoffmann-La Roche, Genmab, GSK, ImmunoGen, Itheos, MSD, Mersana Therapeutics, Myriad Genetics, Novocure, OncoXerna Therapeutics Inc., PharmaMar, Regeneron, Sattucklabs, Seagen/Pfizer, Sutro Biopharma, and Zentalis. CS reports grants from AX'Consulting, AX's Consulting SARL, AstraZeneca, Boehringer Ingelheim, Bristol Myers Squibb, Byondis BV, Daiichi Sankyo, Eisai, Exeter Pharma, F. Hoffmann-La Roche Ltd, Genentech, Grupo de Mama Alemán, Galaad, Glaxo, INDITEX, ISSECAM, Innoup, Grupo Internacional de Estudio del Cáncer de Mama (IBCSG), Liriomacrogénicos, MediTech, Consultoría de Estadísticas Médicas, Medseñor, Menarini, Merck Sharp & Dohme, Merus, Milenio, Instituto Holandés del Cáncer (NKI), Novartis, Pfizer, Philips, Pierre Fabre, Pintafarma, Puma, Queen Mary (University of London), Roche Farma, SACE Medhealth SL, Sanofi, Sanofi Aventis, Seagen, Genética de Seattle, Simon Kucher & Partners SL, Solti, Biofarmacéuticos Synthon, and Zymeworks, consulting fees from AstraZeneca, Daiichi Sankyo, Eisai Europe Ltd., Gilead, Novartis, Pharmalex, Pfizer Inc., Philips Health Works, Pierre Fabre, PintPharma, Puma Biotechnology Inc., Roche Farma SA, Seagen International, Solti, Synthon Biopharmaceuticals, and Zymeworks, payment or honoraria for lectures from AstraZeneca, Daiichi Sankyo, Eisai Europe Ltd., Gilead, Novartis, Pharmalex, Pfizer Inc., Philips Health Works, Pierre Fabre, PintPharma, Puma Biotechnology Inc., Roche Farma SA, Seagen International, Solti, Synthon Biopharmaceuticals, Zymeworks, Sociedade Portuguesa de Oncología, travel aid from AstraZeneca, Daiichi Sankyo, Gilead, Lilly, Novartis, Pfizer Inc., Pierre Fabre, PintPharma, Roche Farma SA, Seagen International, Solti, participation on an advisory board for AstraZeneca, Daiichi Sankyo, Gilead, Lilly, Novartis, Pfizer Inc., Pierre Fabre, PintPharma, Roche Farma SA, Seagen, and research funding in the form of third-party medical writing support, furnished by Eleanor Porteous, MSc, of Nucleus Global, an Inizio Company, was provided by F. Hoffmann-La Roche Ltd. EE reports consulting fees from Amgen, Bayer, Cure Teq, AG Hoffmann-La Roche, BMS, Boehringer Ingelheim, Janssen, Lilly, Medscape, Merck Serono, MSD, Novartis, Organon, Pfizer, Pierre Fabre, Repare Therapeutics, RIN Institute, Sanofi, payment or honoraria for lectures from Amgen, Bayer, Cure Teq, AG Hoffmann-La Roche, BMS, Boehringer Ingelheim, Janssen, Lilly, Medscape, Merck Serono, MSD, Novartis, Organon, Pfizer, Pierre Fabre, Repare Therapeutics Inc., RIN Institute Inc., Sanofi, Seagen, Servier and Takeda, travel aid from Amgen, Bayer, Cure Teq, AG Hoffmann-La Roche, BMS, Boehringer Ingelheim, Janssen, Lilly, Medscape, Merck Serono, MSD, Novartis, Organon, Pfizer, Pierre Fabre, Repare Therapeutics Inc., RIN Institute Inc., Sanofi, Seagen, Servier and Takeda, and participation on Advisory Board for Amgen, Bayer, Cure Teq, AG Hoffmann-La Roche, BMS, Boehringer Ingelheim, Janssen, Lilly, Medscape, Merck Serono, MSD, Novartis, Organon, Pfizer, Pierre Fabre, Repare Therapeutics, RIN Institute, Sanofi, Seagen, Servier and Takeda. EF reports consulting fees from AbbVie, Amgen, AstraZeneca, Bayer, BeiGene, Boehringer Ingelheim, Bristol Myers Squibb, Eli Lilly, F. Hoffmann-La Roche, Genmab, Gilead, GlaxoSmithKline, Janssen, Merck Serono, Merck Sharp & Dohme, Novartis, Peptomyc, Pfizer, Regeneron, Sanofi, Takeda, Turning Point, and Daiichi Sankyo; payment or honoraria for lectures from Amgen, AstraZeneca, Bristol Myers Squibb, Daiichi Sankyo, Eli Lilly, F. Hoffmann-La Roche, Genentech, Janssen, Medical Trends, Medscape, Merck Serono, Merck Sharp & Dohme, PeerVoice, Pfizer, Sanofi, Takeda, and Touch Oncology; payment for expert testimony from AstraZeneca, Janssen, and Roche; and is an independent member of the board for Grifols. EG reports grants from Novartis, Roche, Thermo Fisher, AstraZeneca, Taiho, BeiGene, and Janssen; consulting fees from Roche, Ellipses Pharma, Boehringer Ingelheim, Janssen Global Services, Seattle Genetics, Thermo Fisher, MabDiscovery, Anaveon, F-Star Therapeutics, Hengrui, Sanofi, Incyte, Medscape, Pfizer, and Amgen; payment or honoraria for lectures from Merck Sharp & Dohme, Roche, Thermo Fisher, Novartis, and SeaGen; employment for NEXT Oncology; and serves as a principal investigator for Adaptimmune LLC, Affimed GmbH, Amgen SA, Anaveon AG, AstraZeneca AB, Bicycletx Ltd, BioInvent International AB, Biontech SE, Biontech Small Molecules GmbH, Boehringer Ingelheim International GmbH, Catalym GmbH, Cyclacel Biopharmaceuticals, Cytovation AS, Cytomx, F. Hoffmann-La Roche Ltd, F-Star Beta Limited, Genentech Inc, Genmab B.V., Hifibio Therapeutics, Hutchison Medipharma Limited, Icon, Imcheck Therapeutics, Immunocore Ltd, Incyte Corporation, Incyte Europe Sàrl, Janssen-Cilag International NV, Janssen-Cilag SA, Laboratorios Servier SL, Medimmune LLC, Merck & Co., Inc., Merck KGaA, Novartis Farmacéutica S.A., Peptomyc, Pfizer Slu, Relay Therapeutics, Replimmune, Ribon Therapeutics, Ryvu Therapeutics SA, Seattle Genetics Inc., Sotio AS, Sqz Biotechnologies, Symphogen A/S, Taiho Pharma USA Inc., and T-Knife GmbH. JT reports consulting fees from Alentis Therapeutics, AstraZeneca, Aveo Oncology, Boehringer Ingelheim, Cardiff Oncology, CARSgen Therapeutics, Chugai, Daiichi Sankyo, F. Hoffmann-La Roche Ltd, Genentech Inc, hC Bioscience, Ikena Oncology, Immodulon Therapeutics, Inspirna Inc, Lilly, Menarini, Merck Serono, Merus, MSD, Mirati, Neophore, Novartis, Ona Therapeutics, Orion Biotechnology, Peptomyc, Pfizer, Pierre Fabre, Samsung Bioepis, Sanofi, Scandion Oncology, Scorpion Therapeutics, Seattle Genetics, Servier, Sotio Biotech, Taiho, Takeda Oncology, and Tolremo Therapeutics; payment or honoraria from Medscape Education, PeerView Institute for Medical Education, and Physicians' Education Resource (PER); and stocks in Oniria Therapeutics, Alentis Therapeutics, Pangaea Oncology, and 1TRIALSP. DP reports grants from Merck; consulting fees from Amgen, Ipsen, and Esteve; payment or honoraria for lectures from Amgen, Novartis, and BMS; and travel aid from Amgen, Merck, Roche, Lilly, Servier, Sanofi, and Ipsen. RD reports grants from Merck, Novartis, Daiichi-Sankyo, GlaxoSmithKline, and AstraZeneca; consulting fees from Roche, Foundation Medicine, and AstraZeneca; payment or honoraria for lectures from Roche, Ipsen, Amgen, Servier, Sanofi, Libbs, Merck Sharp & Dohme, Lilly, AstraZeneca, Janssen, Takeda, Bristol-Myers Squibb, GlaxoSmithKline, and Gilead; and holds stocks in Trialing Health. JC reports consulting fees from Astellas Pharma, AstraZeneca, Bayer, Bristol-Myers Squibb, Exelixis, Ipsen, Johnson & Johnson, MSD Oncology, Novartis (AAA), Pfizer, Sanofi, payment or honoraria for lectures from Astellas Pharma, Bayer, Johnson & Johnson, Sanofi, and support for attending meetings from BMS, Ipsen, Roche, AstraZeneca, and Bayer. Disclosures have been attached in a separate document by each author. More detailed disclosures can be found in the ICMJE forms uploaded. All other authors declare no conflicts of interest.
