# Supplementary material for: Development of a prognostic model to predict 90-day mortality in hospitalised cancer patients (PROMISE tool): a prospective observational study
Source: Lancet Reg Health Eur. 2024 Oct 9;46:101063. doi: 10.1016/j.lanepe.2024.101063 (PMC11551497; doi:10.1016/j.lanepe.2024.101063)
Supplement: Supplementary Figures S1–S5 [file mmc1.pdf]

A

Linear predictor formula of the final model

Y= -1.5783+
Polymorphonuclear cells (*x10^9/L)*0.0499 +
Polymorphonuclear cells (*x10^9/L)’*0.0010^3 +
LDH (UI/L)*0.0046 +
LDH (UI/L)’*-0.0089^3 +
Albumin (g/dL)*-0.6165 +
Albumin (g/dL)’*-0.3758^3 +
If(ECOG = 2-3-4: 0.6623) +
If(ECOG = 0: 0) +
If(Last response = PD: 1.8577) +
If(Last response = SD: 1.3014) +
If(Last response = CR-PR: 0)

Probability of risk calculation: exp(Y)/(1+exp(Y))

B

CUTOFFS

Low Risk
<27.33%

Intermediate Risk
≥27.33% & ≤53.04%

High Risk
>53.04%

Supplementary Figure 1: Formula of the final PROMISE score (A), and cutoffs for risk groups (B).
Abbreviations: LDH: lactic acid dehydrogenase; cR: complete response; PR: partial response; SD: stable disease; PD: progressive disease.

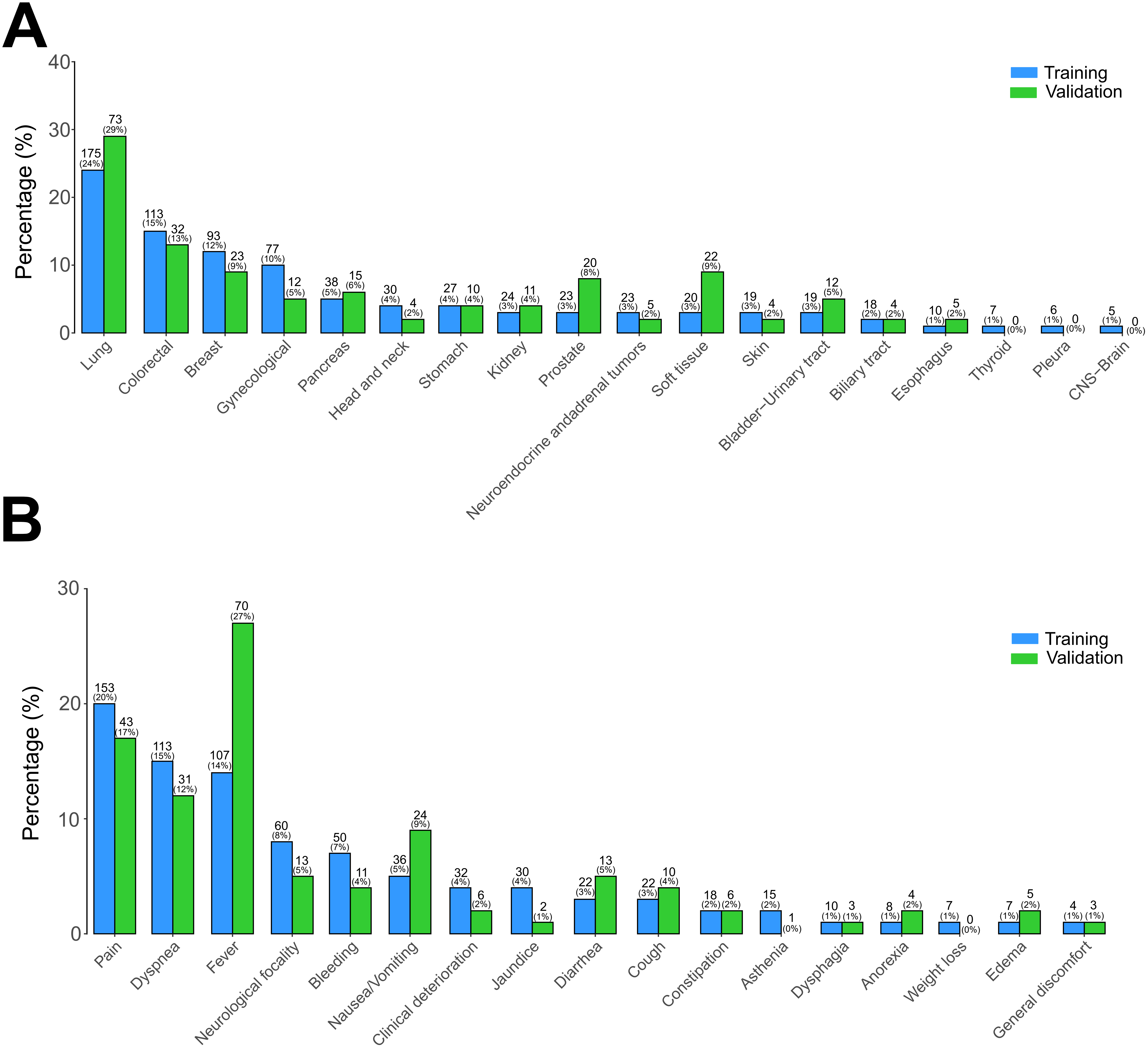

Supplementary Figure 2: Tumor type (A) and reason for unplanned hospital admission (B), in the training (blue) and validation cohorts (green). Abbreviations: CNS: central nervous system.

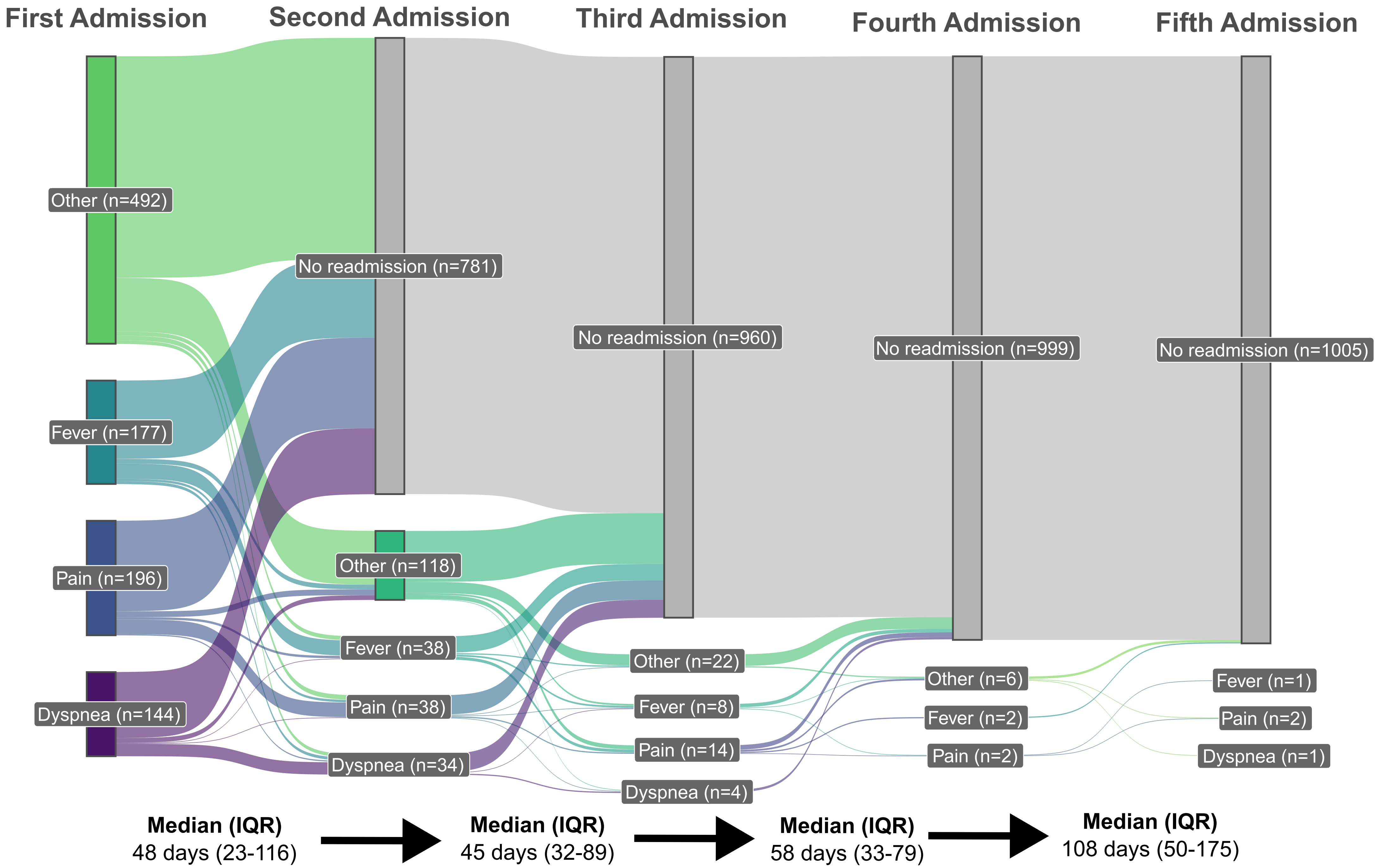

Supplementary Figure 3: Sankey plot depicting total admissions and median time (IQR) between consecutive admissions.
Abbreviations: IQR: interquartile range; n: sample size.

	N	OR (95% CI)	p.value
**Age**	749	1.00 (0.99, 1.01)	0.66
**Sex**			
Male	369	Reference	
Female	380	1.13 (0.85, 1.52)	0.40
**Charlson Comorbidity Index**	749	1.06 (0.97, 1.15)	0.19
**Number of lines**	749	1.16 (1.06, 1.26)	<0.001
**BMI**	749	0.89 (0.69, 1.16)	0.40
**Leucocytes (*x10^9/L) (10 units increase)**	749	1.91 (1.46, 2.52)	<0.001
**Polymorphonuclear cells (*x10^9/L) (10 units increase)**	749	2.29 (1.68, 3.15)	<0.001
**Lymphocytes (*x10^9/L) (10 units increase)**	749	0.33 (0.05, 0.98)	0.25
**Sodium (mmol/L)**	749	0.98 (0.95, 1.01)	0.26
**LDH (UI/L) (1000 units increase)**	749	2.33 (1.66, 3.47)	<0.001
**Albumin (g/dL)**	749	0.41 (0.32, 0.54)	<0.001
**PCR (mg/dL) (100 units increase)**	749	11.35 (2.93, 46.18)	<0.001
**ECOG PS**			
0−1	428	Reference	
2−3−4	321	2.74 (2.03, 3.70)	<0.001
**High Blood Pressure**			
No	475	Reference	
Yes	274	0.93 (0.69, 1.26)	0.66
**Stage**			
I−II−III	266	Reference	
IV	483	1.26 (0.93, 1.71)	0.14
**Dyslipidemia**			
No	528	Reference	
Yes	221	0.96 (0.70, 1.32)	0.79
**Current Treatment**			
Chemotherapy	417	Reference	
Immunotherapy	160	0.99 (0.69, 1.43)	0.97
Other	64	1.26 (0.74, 2.14)	0.39
Targeted therapy	108	0.70 (0.45, 1.08)	0.11
**Oncologic treatment response**			
CR−PR	141	Reference	
PD	444	6.54 (4.05, 11.05)	<0.001
SD	164	2.96 (1.71, 5.30)	<0.001
**Thrombosis**			
No	703	Reference	
Yes	46	1.29 (0.71, 2.35)	0.40
**Clinical trial enrollment**			
No	433	Reference	
Yes	316	0.72 (0.54, 0.97)	0.03
**Primary tumour**			
Breast	93	Reference	
Colorectal	113	1.85 (1.06, 3.27)	0.03
Gynecological	77	1.60 (0.86, 2.97)	0.14
Lung	175	1.27 (0.76, 2.15)	0.37
Other	230	0.99 (0.60, 1.65)	0.96
Pancreas	38	2.79 (1.29, 6.17)	0.01
Prostate	23	1.98 (0.79, 5.06)	0.15
**Smoker habits**			
Never	389	Reference	
Current/Former	360	0.72 (0.54, 0.96)	0.03

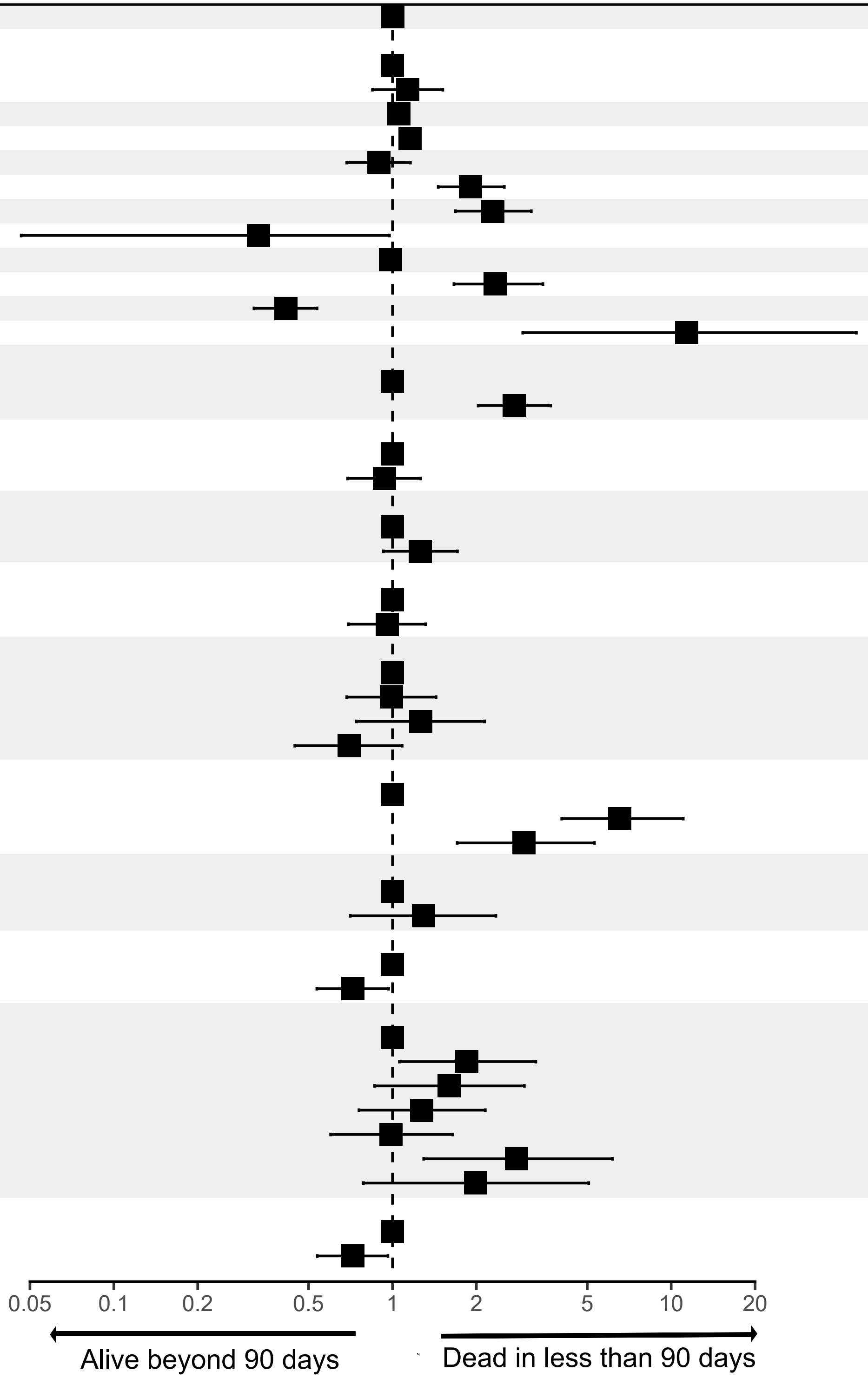

Supplementary Figure 4: Univariate analysis of PROMISE score variables influencing 90-day mortality after unplanned hospitalization in the training cohort.
Abbreviations: BMI: body mass index; LDH: lactic acid dehydrogenase; PCR: polymerase chain reaction; CR: complete response; PR: partial response; SD: stable disease; PD: progressive disease; N: sample size; OR: odds ratio; CI: confidence interval.

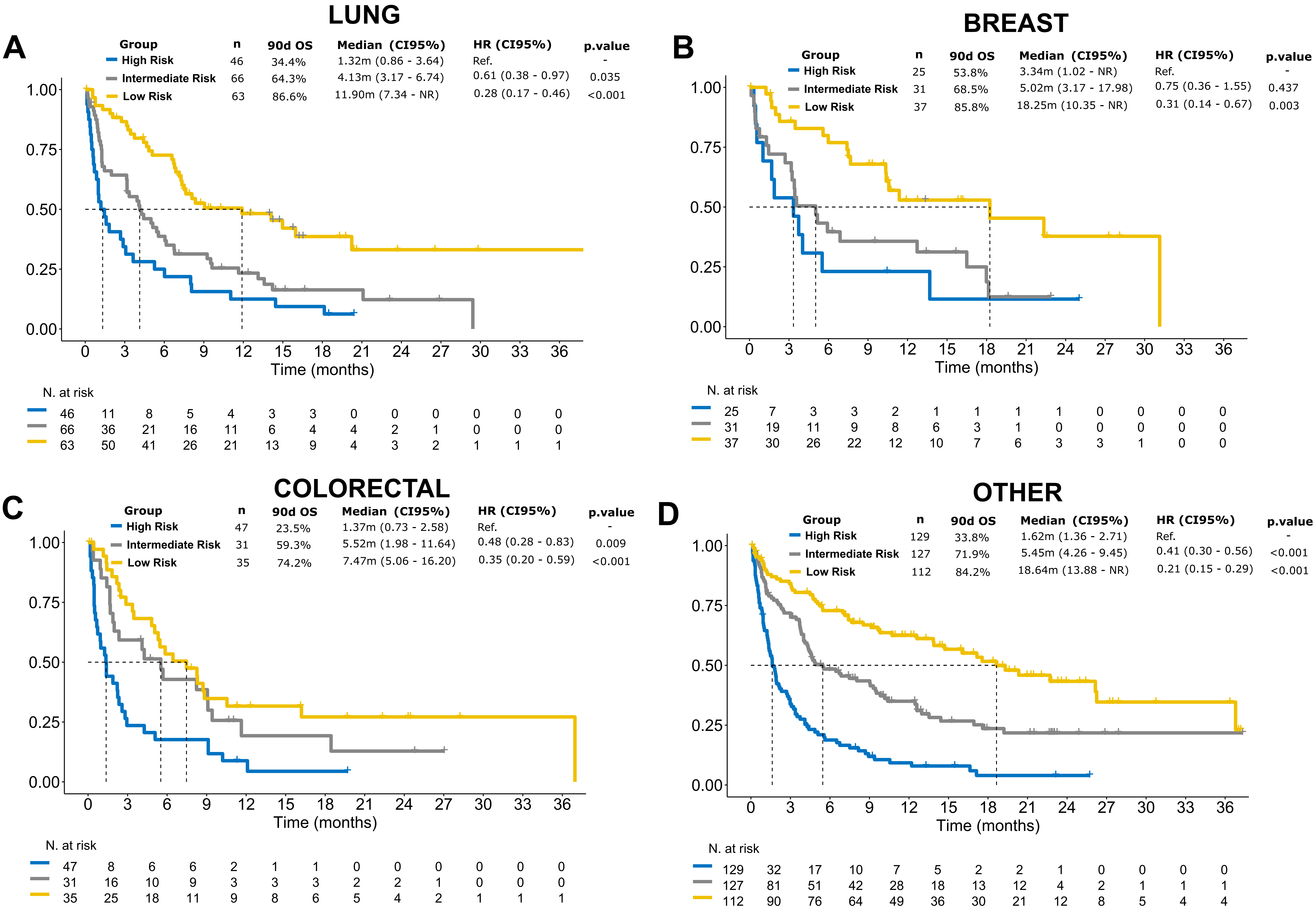

Supplementary Figure 5: Kaplan-Meier curves for overall survival illustrate the 90-day survival rates in the training cohort, stratified by risk groups (high, intermediate, or low PROMISE score) for lung (A), breast (B), colorectal (C), and other tumor types (D).
Abbreviations: n: sample size; 90d OS: 90d percentage of Overall Survival; CI: confidence interval; HR: hazard ratio.
