# Supplementary material for: Development of a prognostic model to predict 90-day mortality in hospitalised cancer patients (PROMISE tool): a prospective observational study
Source: Lancet Reg Health Eur. 2024 Oct 9;46:101063. doi: 10.1016/j.lanepe.2024.101063 (PMC11551497; doi:10.1016/j.lanepe.2024.101063)
Supplement: Supplementary Tables S1–S3 [file mmc2.docx]

**Supplementary Tables**

**Supplementary Table 1:** Missing and imputed values from the training cohort.

|  | Training Cohort | | |
| --- | --- | --- | --- |
|  | **Not imputed** | **Imputed** | **Missings** |
| ECOG |  |  | 0 (0%) |
| - Ecog 0-1 | 428 (57.14%) | 428 (57.14%) |  |
| - Ecog 2-3-4 | 321 (42.86%) | 321 (42.86%) |  |
| Smoker |  |  | 0 (0%) |
| - Never | 389 (51.94%) | 389 (51.94%) |  |
| - Current/Former | 360 (48.06%) | 360 (48.06%) |  |
| Dislypidemia |  |  | 0 (0%) |
| - No | 528 (70.49%) | 528 (70.49%) |  |
| - Yes | 221 (29.51%) | 221 (29.51%) |  |
| High Blood Pressure |  |  | 0 (0%) |
| - No | 475 (63.42%) | 475 (63.42%) |  |
| - Yes | 274 (36.58%) | 274 (36.58%) |  |
| Stage |  |  | 34 (4.54%) |
| - I-II-III | 249 (34.83%) | 266 (35.51%) |  |
| - IV | 466 (65.17%) | 483 (64.49%) |  |
| Current treatment |  |  | 15 (2%) |
| - Chemotherapy | 411 (55.99%) | 417 (55.67%) |  |
| - Immunotherapy | 155 (21.12%) | 160 (21.36%) |  |
| - Other | 61 (8.31%) | 64 (8.54%) |  |
| - Targeted therapy | 107 (14.58%) | 108 (14.42%) |  |
| Enrollment in a clinical trial |  |  | 0 (0%) |
| - No | 433 (57.81%) | 433 (57.81%) |  |
| - Yes | 316 (42.19%) | 316 (42.19%) |  |
| Last response |  |  | 185 (24.7%) |
| - CR-PR | 102 (18.09%) | 141 (18.83%) |  |
| - PD | 343 (60.82%) | 444 (59.28%) |  |
| - SD | 119 (21.1%) | 164 (21.9%) |  |
| Thrombosis |  |  | 1 (0.13%) |
| - No | 703 (93.98%) | 1. 3.86%) |  |
| - Yes | 45 (6.02%) | 46 (6.14%) |  |
| Sex |  |  | 0 (0%) |
| - Male | 369 (49.27%) | 369 (49.27%) |  |
| - Female | 380 (50.73%) | 380 (50.73%) |  |
| Primary tumor type |  |  | 0 (0%) |
| - Breast | 93 (12.42%) | 93 (12.42%) |  |
| - Colorectal | 113 (15.09%) | 113 (15.09%) |  |
| - Gynecological | 77 (10.28%) | 77 (10.28%) |  |
| - Lung | 175 (23.36%) | 175 (23.36%) |  |
| - Other | 230 (30.71%) | 230 (30.71%) |  |
| - Pancreas | 38 (5.07%) | 38 (5.07%) |  |
| - Prostate | 23 (3.07%) | 23 (3.07%) |  |
| Age |  |  | 1 (0.13%) |
| - median (range) | 65 (56 - 72) | 65 (56 - 72) |  |
| - mean (SD) | 63.63 (12.14) | 63.64 (12.13) |  |
| Number of lines |  |  | 13 (1.74%) |
| - median (range) | 2 (1 - 3) | 2 (1 - 3) |  |
| - mean (SD) | 2.37 (1.76) | 2.378 (1.76) |  |
| BMI |  |  | 45 (6.01%) |
| - median (range) | 24.3 (21.3 - 27.7) | 24.3 (21.3 - 27.7) |  |
| - mean (SD) | 24.61 (5.57) | 24.56 (5.56) |  |
| Leucocytes (*x10^9/L) |  |  | 2 (0.27%) |
| - median (range) | 8.03 (5.19 - 11.66) | 8.03 (5.19 - 11.62) |  |
| - mean (SD) | 9.012 (5.83) | 9.007 (5.82) |  |
| Polymorphonuclear cells (*x10^9/L) | |  | 2 (0.27%) |
| - median (range) | 6 (3.6 - 9.4) | 6 (3.6 - 9.4) |  |
| - mean (SD) | 7.054 (5.22) | 7.046 (5.22) |  |
| Lymphocytes (*x10^9/L) |  |  | 2 (0.27%) |
| - median (range) | 0.9 (0.5 - 1.3) | 0.9 (0.5 - 1.3) |  |
| - mean (SD) | 2.507 (40.22) | 2.503 (40.16) |  |
| Sodium (mmol/L) |  |  | 7 (0.93%) |
| - median (range) | 136.1 (133.5 - 138.3) | 136.1 (133.5 - 138.3) |  |
| - mean (SD) | 135.4 (5.41) | 135.4 (5.42) |  |
| LDH (UI/L) |  |  | 102 (13.62%) |
| - median (range) | 268 (203.5 - 401) | 268 (204 - 405) |  |
| - mean (SD) | 493.4 (962.4) | 492.3 (929.61) |  |
| Albumin (g/dL) |  |  | 68 (9.08%) |
| - median (range) | 3.3 (2.8 - 3.7) | 3.3 (2.8 - 3.7) |  |
| - mean (SD) | 3.271 (0.6) | 3.277 (0.61) |  |
| PCR (mg/dL) |  |  | 62 (8.28%) |
| - median (range) | 7.65 (1.74 - 16.3) | 7.65 (1.75 - 16.28) |  |
| - mean (SD) | 11.02 (13.99) | 10.91 (13.64) |  |

**Supplementary Table 2:** Most common symptoms causing hospital admission and the diagnoses causing the symptoms.

| **Symptom causing admission** | **Training Cohort** | | | | | | **Validation Cohort** | | | |
| --- | --- | --- | --- | --- | --- | --- | --- | --- | --- | --- |
|  | **N patients^1^** | | **Diagnosis**  **(Number of patients; %)** | | | | **N patients^1^** | **Diagnosis**  **(Number of patients; %)** | | |
| Pain | 153 | | Disease progression/ Cancer-related pain (52; 34%) | Intestinal sub-occlusion/ occlusion  (23; 15%) | | Infection^2^  (8; 5.2%) | 43 | Disease progression/ Cancer-related pain  (12; 27.9%) | Infection^2^  (8; 18.6%) | Intestinal sub-occlusion/ occlusion  (3; 6.9%) |
| Fever | 107 | | Infection^2^  (56; 52.3%) | Febrile neutropenia (14; 13.1%) | | Fever of unknown origin (5; 4.7%) | 70 | Infection^2^  (32; 45.7%) | Febrile neutropenia  (7; 10.0%) | Septic shock  (6; 8.6%) |
| Dyspnea | 65 | | Pleural effusion  (24; 21.2%) | Infection^2^  (24; 21.2%) | | Disease progression  (17; 15%) | 22 | Infection^2^  (11; 35.5%) | Pleural effusion  (7; 22.6%) | Thromboembolic disease  (4; 12.9%) |
| Bleeding^3^ | 50 | | Disease progression (19; 38.0%) | Gastrointestinal Ulcer  (7; 14.0%) | | Treatment toxicity^4^  (7; 14%) | 9 | Disease progression (5; 55.5%) | Gastrointestinal Ulcer  (1; 11.1%) | Treatment toxicity^4^  (1; 11.1%) |
| Neurological focality | 30 | | Disease progression (16; 26.7%) | Spinal cord compression (9; 15%) | | Neurological disease^5^  (5; 8.3%) | 9 | Neurological disease^5^  (4; 30.8%) | Metastasis  (3; 23.1%) | Spinal cord compression (2; 15.4%) |
| Nausea/ vomiting | 25 | Intestinal sub-occlusion/ occlusion (18; 50.0%) | | | Chemotherapy-related toxicity (4; 11.1%) | Disease progression  (3; 8.3%) | 13 | Intestinal sub-occlusion/ occlusion  (7; 29.2%) | Infection^2^  (4; 16.7%) | Chemotherapy-related toxicity (2; 8.3%) |
| **Global** | 430 | Infection^2^ (134; 17.9%) | | | Disease progression (99; 13.2%) | Intestinal sub-occlusion/occlusion (49; 6.5%) | 165 | Infection^2^  (67; 25.8%) | Disease progression (21; 8.1%) | Respiratory failure  (17; 6.5%) |

1. Number (N) of patients admitted for the given symptom, and for which a diagnosis was determined.
2. Infection in any tissue (abdominal, respiratory, intravascular, skin, soft tissue, etc.) and of any origin (bacteria, virus, fungi, parasite, or mycobacteria).
3. Bleeding includes hematemesis, melena, hemoptysis, hematochezia, etc.
4. Treatment toxicity: chemotherapy, targeted therapy or immunotherapy related.
5. Neurological disease diagnosed other than stroke (dementia, epilepsy, encephalopathy, migraine, etc.).

**Supplementary Table 3: PROMISE score metrics per tumor type in both training and validation cohorts.**

|  |  | Accuracy | PPV | NPV | Specificity | Sensitivity | AUC (95% CI) |
| --- | --- | --- | --- | --- | --- | --- | --- |
| Training | **Lung** (n=175) | 0.72 | 0.72 | 0.86 | 0.81 | 0.79 | 0.78 (0.71 – 0.85) |
|  | **Breast** (n=93) | 0.75 | 0.72 | 0.86 | 0.82 | 0.78 | 0.81 (0.71 – 0.90) |
|  | **Colorectal** (n=113) | 0.74 | 0.79 | 0.80 | 0.75 | 0.84 | 0.82 (0.75 – 0.90) |
|  | **Other** (n=368) | 0.73 | 0.71 | 0.85 | 0.75 | 0.82 | 0.79 (0.72 – 0.85) |
| Validation | **Lung** (n=73) | 0.79 | 0.68 | 0.88 | 0.77 | 0.83 | 0.76 (0.65 – 0.88) |
|  | **Breast** (n=23) | 0.71 | 0.60 | 0.75 | 0.82 | 0.50 | 0.64 (0.40 – 0.89) |
|  | **Colorectal** (n=32) | 0.93 | 0.88 | 1.00 | 0.88 | 1.00 | 0.82 (0.67 – 0.97) |
|  | **Other** (n=132) | 0.77 | 0.80 | 0.75 | 0.80 | 0.75 | 0.79 (0.69 – 0.89) |
